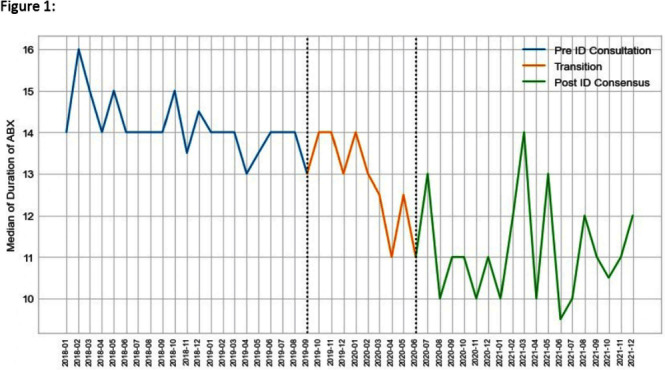# Infectious Diseases Consultation Reduces Antibiotic Duration for Uncomplicated Gram-Negative Bacteremia

**DOI:** 10.1017/ash.2024.106

**Published:** 2024-09-16

**Authors:** Mary Acree, Sandra Naegele, Bianca Baik, Urmila Ravichandran, Nirav Shah

**Affiliations:** NorthShore University HealthSystem; Endeavor Health

## Abstract

**Background:** Unlike Staphylococcus aureus bacteremia, the impact of ID consultation for gram negative bacteremia (GNB) has not been well studied. Recent literature has supported shorter courses of antibiotics in adults with uncomplicated GNB. We examined duration of therapy for adult patients with uncomplicated GNB over a period of time during which ID consultation for GNB was made mandatory. **Method:** NorthShore University HealthSystem is a 4 hospital, 828 bed hospital system located in the northern suburbs of Chicago. Data were collected retrospectively from 1/1/2018 through 12/31/2021 for patients 18 years or older hospitalized with an uncomplicated bloodstream infection due to Escherichia coli, Klebsiella species or Proteus species. This study was approved by the Institutional Review Board. All sources of infection were included. Days of effective antibiotic therapy were extracted manually by two pharmacists and an infectious diseases physician. During the study period, two major changes occurred: 1) the Physician Practice Council of NorthShore University Healthsystem voted in favor of a mandatory ID consultation for GNB and 2) the ID division developed a treatment algorithm for GNB management with an emphasis on shorter antibiotic duration in uncomplicated cases. This study was divided into three time periods: Pre-ID consultation – 1/1/2018 – 8/31/2019; Transition – 9/1/2019 – 5/31/2020 (after mandatory ID consultation, before ID division consensus achieved); Post-ID Consensus – 6/1/2020 – 12/31/21. Primary outcome was duration of antibiotic therapy. Secondary outcomes included in-hospital all-cause mortality and 30 day readmission. Continuous variables were described using median and interquartile range, and categorical data using frequency and prevalence. Kruskal-Wallis rank sum test for continuous and χ2 for categorical variables was used to verify similarity among the pre-ID consultation, transition and post-ID consensus periods. The analysis was performed using Python. **Result:** 1026 patients were included in the study. Pathogens included 773 E. coli (75.4%), 193 Klebsiella species (18.8%) and 60 Proteus species (5.9%). Length of stay, 30 day readmission and in-hospital mortality were not statistically significantly different when comparing pre-ID consultation and post-ID consensus time periods. Total duration of therapy was statistically significantly shorter in the post-ID consensus period (p < 0.001). **Conclusion:** Mandatory ID consultation and development of an ID consensus approach can shorten antibiotic duration in uncomplicated GNB. Further analysis will explore timing of transition to oral therapy and syndromic differences.

**Figure:**
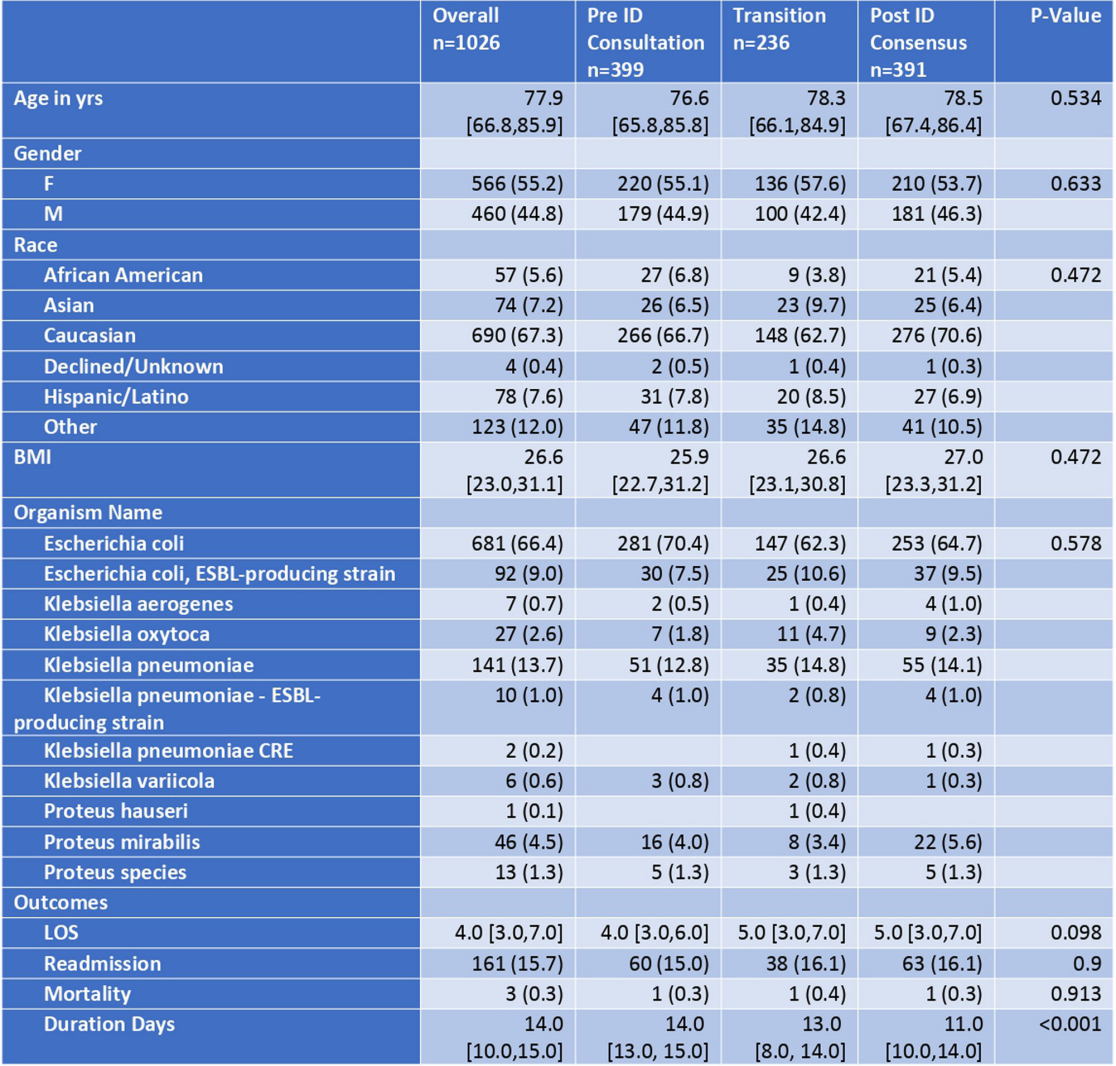

**Figure:**